# Mechanistic aspects of biosynthesis of silver nanoparticles by several *Fusarium oxysporum *strains

**DOI:** 10.1186/1477-3155-3-8

**Published:** 2005-07-13

**Authors:** Nelson Durán, Priscyla D Marcato, Oswaldo L Alves, Gabriel IH De Souza, Elisa Esposito

**Affiliations:** 1 Biological Chemistry Laboratory, Instituto de Química, Universidade Estadual de Campinas, CEP 13084862, Caixa Postal 6154, Campinas, S.P., Brazil; 2 Biological Chemistry and Biotechnology Laboratory, Center Environmental Sciences, Universidade de Mogi das Cruzes, Mogi das Cruzes, S.P., Brazil; 3 Solid State Chemistry Laboratory, Instituto de Química, Universidade Estadual de Campinas, CEP 13084862, Caixa Postal 6154, Campinas, S.P., Brazil

## Abstract

Extracellular production of metal nanoparticles by several strains of the fungus *Fusarium oxysporum *was carried out. It was found that aqueous silver ions when exposed to several *Fusarium oxysporum *strains are reduced in solution, thereby leading to the formation of silver hydrosol. The silver nanoparticles were in the range of 20–50 nm in dimensions. The reduction of the metal ions occurs by a nitrate-dependent reductase and a shuttle quinone extracellular process. The potentialities of this nanotechnological design based in fugal biosynthesis of nanoparticles for several technical applications are important, including their high potential as antibacterial material.

## Background

The dissimilatory ferric reductase, which are found in bacteria are an essential part of the iron cycles [1] and are essentially intracellular, but one extracellular one was isolated from *Mycobacterium paratuberculosis *[2]. Another possible mechanism could be active in this process since it was discovered that some bacteria reduce Fe^3+ ^oxides by producing and secreting small, diffusible redox compounds that can serve as electron shuttle between the microbe and the insoluble iron substrate [3]. The role of excreted compounds in extracellular electron transfer was recently reviewed [4].

The presence of hydrogenase in fungus as *Fusarium oxysporum *was demonstrated with washed cell suspensions that had been grown aerobically and anaerobically in a medium with glucose and salts amended with nitrate [5]. The nitrate reductase was apparently essential for ferric iron reduction [6]. Many fungi that exhibit these characteristic properties, in general, are capable of reducing Au (III) or Ag (I) [7]. Besides these extracellular enzymes, several naphthoquinones [8-10] and anthraquinones [11] with excellent redox properties, were reported in *F. oxysporum *that could be act as electron shuttle in metal reductions [3].

Although it is known that microorganisms such as bacteria, yeast and now fungi play an important role in remediation of toxic metals through reduction of the metal ions, this was considered interesting as nanofactories very recently [12]. Using these dissimilatory properties of fungi, the biosynthesis of inorganic nanomaterials using eukaryotic organisms such as fungi may be used to grow nanoparticles of gold [13] and silver [14] intracellularly in *Verticillium *fungal cells [15]. Recently, it was found that aqueous chloroaurate ions may be reduced extracellularly using the fungus *F. oxysporum*, to generate extremely stable gold [16] or silver nanoparticles in water [17]. Other process, which was described in the literature, was related to produce silver nanoparticles through oligopeptides catalysis, precipitating the particles with several forms (hexagonal, spherical and triangular) [18]. However, in the fungal reduction of Ag ions led colloidal suspension, differently that in the oligopeptides case. Then the mechanistic aspects are still an open question, however this process occur in the fungal case probably either by reductase action or by electron shuttle quinones or both. Our aims in this research are to compare different strains of *F. oxysporum *in order to understand if the efficiency of the reduction of silver ions is related to a reductase or quinone action.

## Results and Discussion

**Figure 1:**
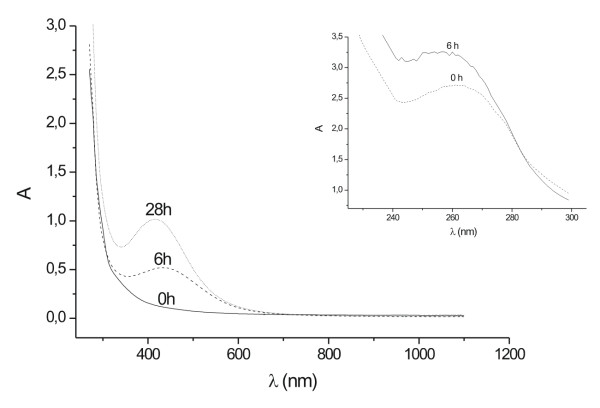
UV-Vis spectra recorded as a function of time of reaction of an aqueous solution of 10^-3 ^M AgNO_3 _with the fungal biomass (07SD). The inset shows the UV-Vis absorption in the low wavelength region.

The Erlenmeyer flasks with the *F. oxysporum *biomass were a pale yellow color before the addition of Ag^+ ^ions and this changed to a brownish color on completion of the reaction with Ag^+ ^ions for 28 h. The appearance of a yellowish-brown color in solution containing the biomass suggested the formation of silver nanoparticles [21]. The UV-Vis spectra recorded from the *F. oxysporum *07SD strain reaction vessels (Method A) at different times of reaction is presented in Figure 1. The strong surface plasmon resonance centered at ca. 415–420 nm clearly increases in intensity with time. The solution was extremely stable, with no evidence of flocculation of the particles even several weeks after reaction. The inset of Figure 1 shows UV-Vis spectra in low wavelength region recorded from the reaction medium exhibited an absorption band at ca. 265 nm and it was attributed to aromatic amino acids of proteins. It is well known that the absorption band at ca. 265 nm arises due to electronic excitations in tryptophan and tyrosine residues in the proteins. This observation indicates the release of proteins into solution by *F. oxysporum *and suggests a possible mechanism for the reduction of the metal ions present in the solution [17].

**Figure 2:**
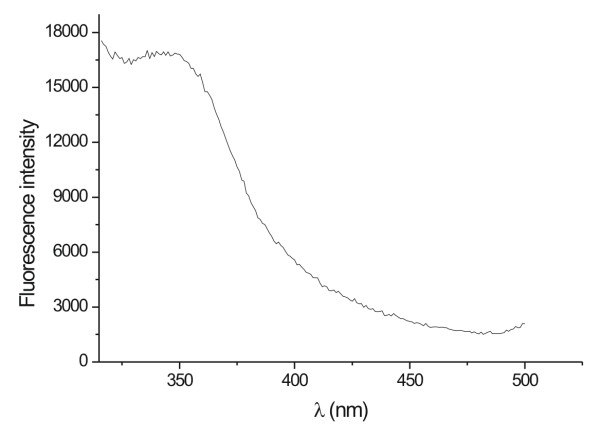
Fluorescence emission spectrum recorded from the silver nanoparticles-fungus reaction mixture. The excitation wavelength was 260 nm.

Figure 2 shows the fluorescence emission spectra of fungal filtrate of one of the strain (07SD). An emission band centered at 340 nm was observed. The nature of the emission band indicates that the proteins bound to the nanoparticle surface and those present in the solution exist in the native form [22]. The similar results were observed for all the studied strains as shown in Table 1. In Table 1, the 07SD strain appeared as the most efficient one in the silver nanoparticles production. Apparently, the different efficiencies are related to the reductase and/or to the quinone generation and will be discussed later. A destabilization of the nanoparticles is evident in the case of *F. oxysporum *534, 9114 and 91248 strains at 28 hrs, as indicated by a decrease in the 420 nm absorption.

**Table 1:** Relative absorbance at 420 nm of several *F. oxysporum *strains

Strains	Relative Absorbance (420 nm)^a^		
	0 hours	6 hours	28 hours

O6 SD	1	2.041	4.854
07 SD	1	4.007	7.922
534	1	1.682	0.712
9114	1	1.686	0.572
91248	1	3.549	1.372

^a^Normalized absorbance.

**Figure 3:**
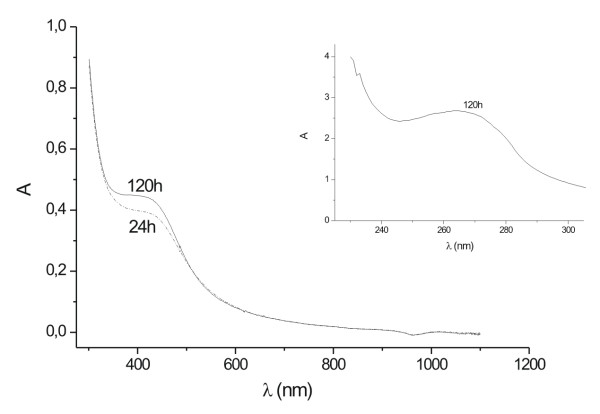
UV-Vis spectra recorded as a function of time of reaction of an aqueous solution of 10^-3 ^M AgNO_3 _with the fungal filtrate (07SD). The inset shows the UV-Vis absorption in the low wavelength region.

Similarly, when the biomass was immersed in water and only the fungal filtrate (Method B) was added to a 10^-3 ^M AgNO_3 _solution, the initially colorless aqueous solution changed to a pale yellowish-brown within 28 h of reaction (data not shown), clearly indicating that the reduction of the ions occurs extracellularly through reducing agents released into the solution by *F. oxysporum *as it shows the UV-Vis spectra for the 07SD strain (Fig. 3).

**Figure 4:**
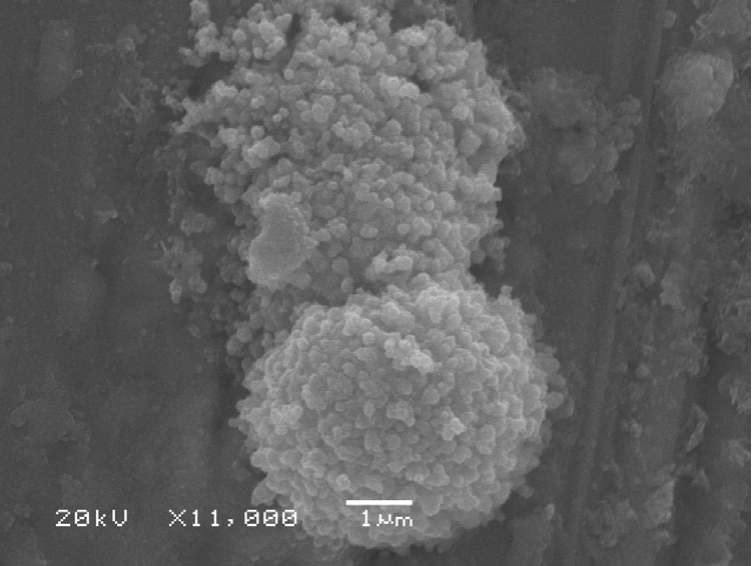
SEM micrograph from *F. oxysporum *07 SD strain at ×11000 magnification.

**Figure 5:**
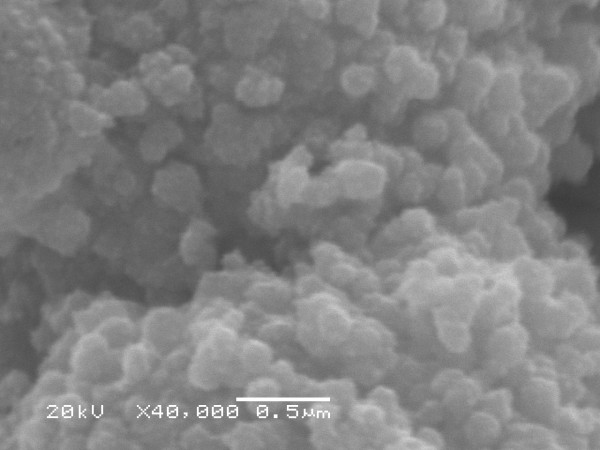
SEM micrograph from *F. oxysporum *07 SD strain at ×40000 magnification.

**Figure 6:**
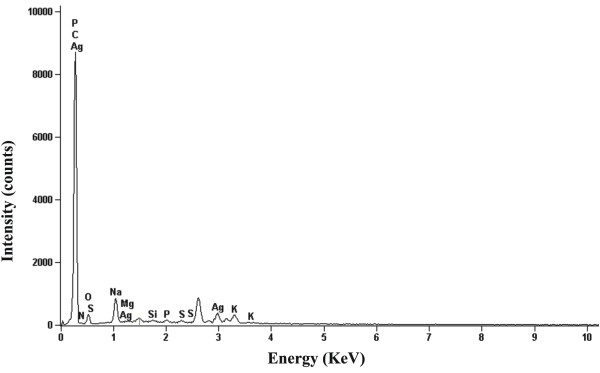
EDS spectra of silver nanoparticles.

Figures 4 and 5 shows the SEM micrograph recorded from the silver nanoparticle (Method A). This picture shows silver nanoparticles aggregates. In this micrograph, spherical nanoparticles in the size range 20–50 nm were observed. The nanoparticles were not in direct contact even within the aggregates, indicating stabilization of the nanoparticles by a capping agent. This corroborates with the previous observation by Ahmad et al. [17] in their study on *F. oxysporum*. The same micrograph in the Method B was observed (not showed). In the analysis by energy dispersive spectroscopy (EDS) of the silver nanoparticles was confirmed the presence of elemental silver signal (Figure 6).

**Figure 7:**
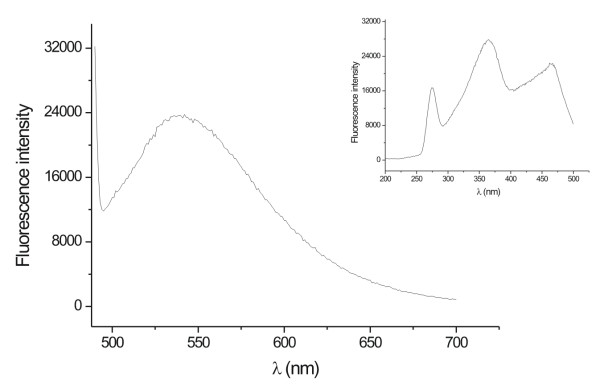
Fluorescence emission spectrum from the aqueous solution of 10^-3 ^M AgNO_3 _with the fungal biomass (07SD). The excitation wavelength was 465 nm. The inset shows the fluorescence excitation spectrum (λ emission at 550 nm).

The TLC (Cromatography of Thin Layer) analysis on silica gel 60 plates using chloroform-methanol-acetic acid (195:5:1) showed a spot with Rf value of 0.65, and using benzene-nitromethane-acetic acid (75:25:2) showed a spot with Rf value of 0.85, corresponding to 2-acetyl-3,8-dihydroxy-6-methoxy anthraquinone or its isomers at 2-acetyl-2,8-dihydroxy-6-methoxy anthraquinone (Scheme 1). This was corroborated by the fluorescence spectrum of the filtrate (Method A), which indicates an anthraquinone fluorescence moiety [11]. The excitation spectra at the maximum emission (550 nm) fit quite well with the absorption spectrum of the anthraquinone in Figure 7.

**Figure 8:**
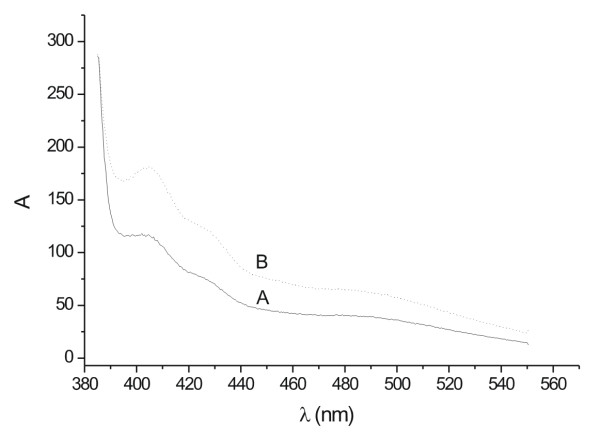
Fluorescence emission spectra for the reaction of nitrite with 2,3-diaminophthalene. In the emission spectra the curves A and B were, respectively: fungal filtrate and fungal filtrate and 0.1% KNO_3 _solution. The maximum excitation wavelength was at 375 nm.

The Figure 8 shows the nitrate reductase through the reaction of nitrite with 2,3-diaminophthalene. The emission spectrum exhibits two major peaks of fluorescence intensity at 405 and 490 nm corresponding to the emission maximum of the and 2,3-diaminonapthotriazole, DAN (excess) respectively. The intensity of these two bands increased with the addition of a 0.1% KNO_3 _solution, confirming the presence of nitrate reductase.

**Figure 9:**
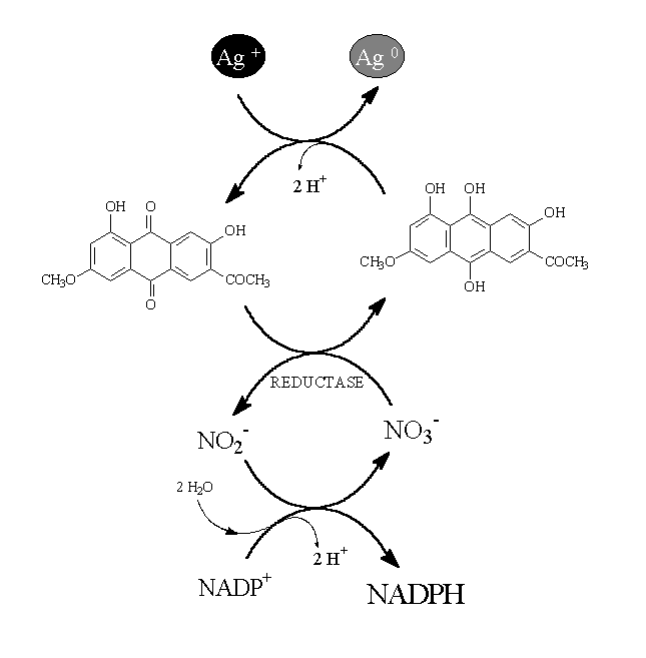
Hypothetical mechanisms of silver nanoparticles biosynthesis.

It appears that the reductase is responsible for the reduction of Ag^+ ^ions and the subsequent formation of silver nanoparticles. The same observation was reported with another strain of *F. oxysporum *and it was pointed out that this reductase was specific to *F. oxysporum*. However, *Fusarium moniliforme*, did not result in the formation of silver nanoparticles, neither intracellularly nor extracellularlybut contained intra and extra cellular reductases in a similar fashion as *F. oxysporum *[17,23]. This is an indication that probably the reductases in this kind of *Fusarium *are important for Fe (III) to Fe (II) but not to Ag (I) to Ag (0). Moreover, in *F. moniliforme *anthraquinones derivatives were not detected unlike the case of *F. oxysporum*. Both *fusarium *were alike in the production of naphthaquinones [8] but differed in the production of anthraquinones. Probably, in our case, Ag (0) reduction was mainly due to a conjugation between the electron shuttle with the reductase participation as shown in Figure 9.

## Conclusion

Even though gold/silver nanoparticles have been synthesized using prokaryotes such as bacteria [24,25] and eukaryotes such as fungi [13,14], the nanoparticles grow intracellularly, except in the case of a recent report in which *F. oxysporum *was used. In that case the nanoparticles grew extracellularly [17]. In our case, all the *F. oxysporum *strains studied exhibited silver nanoparticle production capacity, however, depending on the reductase/electron shuttle relationships under these conditions. Biologically synthesized silver nanoparticles could have many applications, in areas such as non-linear optics, spectrally selective coating for solar energy absorption and intercalation materials for electrical batteries, as optical receptors, catalysis in chemical reactions, biolabelling [26], and as antibacterials capacity [27].

## Methods

The *F. oxysporum *strains used were the following: O6 SD, 07 SD, 534, 9114 and 91248 from ESALQ-USP Genetic and Molecular Biology Laboratory-Piracicaba, S.P., Brazil. The fungal inoculates were prepared in a malt extract 2% and yeast extract 0.5% at 28°C in Petri plates. The liquid fungal growth was carried out in the presence of yeast extract 0.5% at 28°C for 6 days. The biomass was filtrated and resuspended in sterile water.

### Silver reduction and its characterization

Method A: In the silver reduction, the methodology described previously was followed [17]. Briefly, approximately 10 g of *F. oxysporum *biomass was taken in a conical flask containing 100 mL of distilled water. AgNO_3 _solution (10^-3 ^M) was added to the erlenmeyer flask and the reaction was carried out in the dark. Periodically, aliquots of the reaction solution were removed and the absorptions were measured using a UV-Vis spectrophotometer (Agilent 8453 – diode array).

Method B: Another test was also carried out as following: approximately 10 g of *F. oxysporum *biomass was taken in a conical flask containing 100 mL of distilled water, kept for 72 h at 28°C and then the aqueous solution components were separated by filtration. To this solution, AgNO_3 _(10^-3 ^M) was added and kept for several hours at 28°C.

The silver nanoparticles were characterized by scanning electron microscopy (SEM) and energy-dispersive spectroscopy (EDS) at a voltage of 20 kV (Jeol – JSM-6360LV) and previously coated with gold under vacuum.

### Determination of the electron-shuttling compounds

Release of electron-shuttling compounds was followed the methodology described previously [11]: In order to determine the water-soluble quinones that might function as an electron shuttle, cultures were filtered 4–6 weeks, and the filtrate adjusted to pH 3 with HCl 1 M. The acidified solution was then passed through a column with ion exchange resin (Amberlite^®^) for absorption of the pigments. Compounds were removed from the column by elution with acetone, the acetone removed using a Buchi rotary evaporation and the aqueous phase extracted 3 times with ethyl acetate. All ethyl acetate extractions were combined and reduced using the rotary evaporator. After that, 2 μL samples were repeatedly spotted on a Silica gel 60 plate until a spot was visible under UV light at 254 nm. Samples were resolved using a chloroform-methanol-acetic acid (195:5:1) and benzene-nitromethane-acetic acid (75:25:2) system designed to mobilize polar pigments. Plates were air dried, and spots visualized under UV light [19].

### Nitrate reductase assay

Nitrate reduction was demonstrated in the same medium (Method A and B) of the same growth broth of *F. oxysporum *with the addition of 0.1% of KNO_3 _[6]. The nitrate reductase test was made after 2 days by fluorometric method [20]. Briefly, 100 μL fungal filtrate and 200 μL of dionized water. To this, 10 μL of freshly prepared 2,3-diaminonaphtalene (DAN) (0.05 mg/mL in 1 M HCl) is added and mixed immediately. After 10 min incubation at 20°C, the reaction was stopped with 5 μL of 0.1 M NaOH. The intensity of the fluorescent signal produced by the product was maximized by the addition of base. The 2,3-diaminonapthotriazole formation was measured using a Perkin-Elmer (LS-55) luminescence spectrophotometer with and excitation wavelength at 375 nm and the emission band measured at 550 nm [20].

### Determination of the tryptophan/tyrosine residues

Presence of tryptophan/tyrosine residues in proteins release in the fungal filtrated was analyzed by fluorescence [17]. The fluorescence measurements were carried out on a Perkin-Elmer (LS-55) luminescence spectrophotometer. The exitation wavelength was 260 nm, close to maximal optical transitions of the tryptophan and tyrosine.

## Authors' contributions

ND conceived the study, together with OLA and EE and participated in its design and coordination and collected all the data and wrote the paper. PDM obtained all the SEM views, performed the enzymatic assays, the electron shuttling aspects and discussed the three related parts in the manuscript. GIHS performed all the fungal tests and measured all the spectroscopic variations of the plasmon resonance of the silver nanoparticles supervised by EE. OLA also supervised all the nanoparticles aspects in this work. All authors read and approved the final manuscript.
